# Incidence and risk factors for major infections in hospitalized children with nephrotic syndrome

**DOI:** 10.1590/2175-8239-JBN-2019-0001

**Published:** 2019-09-12

**Authors:** Manish Kumar, Jaypalsing Ghunawat, Diganta Saikia, Vikas Manchanda

**Affiliations:** 1 Chacha Nehru Bal Chikitsalaya, Department of Pediatrics and Microbiology, New Delhi, Delhi, India.

**Keywords:** Infection, Peritonitis, Nephrotic Syndrome, Infecção, Peritonite, Síndrome Nefrótica

## Abstract

**Introduction:**

Children with nephrotic syndrome are at increased risk of infections because of disease status itself and use of various immunosuppressive agents. In majority, infections trigger relapses requiring hospitalization with increased risk of morbidity and mortality. This study aimed to determine the incidence, spectrum, and risk factors for major infections in hospitalized children with nephrotic syndrome.

**Methods:**

All consecutive hospitalized children between 1-12 years of age with nephrotic syndrome were enrolled in the study. Children with acute nephritis, secondary nephrotic syndrome as well as those admitted for diagnostic renal biopsy and intravenous cyclophosphamide or rituximab infusion were excluded.

**Results:**

A total of 148 children with 162 admissions were enrolled. Incidence of major infections in hospitalized children with nephrotic syndrome was 43.8%. Peritonitis was the commonest infection (24%), followed by pneumonia (18%), urinary tract infection (15%), and cellulitis (14%), contributing with two thirds of major infections. Streptococcus pneumoniae (n = 9) was the predominant organism isolated in children with peritonitis and pneumonia. On logistic regression analysis, serum albumin < 1.5gm/dL was the only independent risk factor for all infections (OR 2.6; 95% CI, 1.2-6; *p* = 0.01), especially for peritonitis (OR 29; 95% CI, 3-270; *p* = 0.003). There were four deaths (2.5%) in our study, all due to sepsis and multiorgan failure.

**Conclusions:**

Infection remains an important cause of morbidity and mortality in children with nephrotic syndrome. As Pneumococcus was the most prevalent cause of infection in those children, attention should be paid to the pneumococcal immunization in children with nephrotic syndrome.

## RESUMO

**Introdução:**

Crianças com síndrome nefrótica apresentam maior risco de infecções devido ao próprio status da doença e ao uso de vários agentes imunossupressores. Em grande parte, as infecções desencadeiam recidivas que exigem hospitalização, com risco aumentado de morbidade e mortalidade. Este estudo teve como objetivo determinar a incidência, o espectro e os fatores de risco para infecções graves em crianças hospitalizadas com síndrome nefrótica.

**Métodos:**

Todas as crianças hospitalizadas consecutivamente entre 1 e 12 anos de idade com síndrome nefrótica foram incluídas no estudo. Crianças com nefrite aguda, síndrome nefrótica secundária, bem como aquelas admitidas para biópsia renal diagnóstica e infusão intravenosa de ciclofosfamida ou rituximabe foram excluídas.

**Resultados:**

Foram cadastradas 148 crianças com 162 internações. A incidência de infecções graves em crianças hospitalizadas com síndrome nefrótica foi de 43,8%. A peritonite foi a infecção mais comum (24%), seguida por pneumonia (18%), infecção do trato urinário (15%) e celulite (14%), contribuindo com dois terços das principais infecções. Streptococcus pneumoniae (n = 9) foi o organismo predominantemente isolado em crianças com peritonite e pneumonia. Na análise de regressão logística, a albumina sérica < 1,5gm / dL foi o único fator de risco independente para todas as infecções (OR 2,6; 95% CI, 1,2-6; *p* = 0,01), especialmente para peritonite (OR 29; IC95% 3 -270, *p* = 0,003). Houve quatro mortes (2,5%) em nosso estudo, todas devido a sepse e falência de múltiplos órgãos.

**Conclusões:**

A infecção continua sendo uma importante causa de morbimortalidade em crianças com síndrome nefrótica. Como o Pneumococo foi a causa mais prevalente de infecção nessas crianças, deve-se atentar para a imunização pneumocócica em crianças com síndrome nefrótica.

## INTRODUCTION

Nephrotic syndrome (NS) is one of the commonest chronic renal diseases in children, characterized by selective proteinuria, hypoalbuminemia, hyperlipidemia, and edema. Majority of cases of nephrotic syndrome are without underlying secondary etiology and termed idiopathic nephrotic syndrome (INS). Based on response to therapy, these cases are further classified as steroid sensitive (SSNS) and steroid resistant nephrotic syndrome (SRNS). More than 50% cases of SSNS show frequent relapses or become steroid-dependent requiring repeated courses of steroid and other immunosuppressive drugs as steroid sparing agent.^1^ SRNS cases, at the other end, are at additional risk of renal failure. Among the important risk factors for infection are urinary loss of immunoglobulins and alternative complement pathway factors B and I, presence of edema, and treatment with prednisolone and other cytotoxic agents.^2^ Peritonitis, pneumonia, urinary tract infection (UTI), cellulitis, meningitis and tuberculosis have been reported as major infections in these children.^3^^-^ 11 Data are limited on incidence and risk factors for major infections in children with NS from north India. This study aimed to estimate the incidence, pattern, and risk factors for major infections in hospitalized children with INS.

## MATERIAL AND METHODS

This prospective observational study was conducted at a tertiary care pediatric hospital in Delhi from June 2014 to December 2015. All consecutive hospitalized children between 1-12 years of age with diagnosis of NS were screened. NS and associated complications were defined as per guidelines from Indian Pediatric Nephrology Group.^12^ Children with NS were admitted in presence of one or more of the following conditions: anasarca, suspected major infections, or hypovolemia. Major infections were defined as those with disseminated or deep seated infections requiring hospitalizations and treatment with parenteral antibiotics, and were the following: peritonitis pneumonia, cellulitis, meningitis, unexplained pyrexia and infective diarrhea.^12^ Children were subjected to complete blood counts, kidney and liver function tests, lipid profile and urine routine microscopic examination. Ascitic and cerebrospinal fluid cytology, biochemistry, and culture were performed in children with suspected peritonitis and meningitis respectively. Chest X-ray and blood and urine culture were performed as and when required. The exclusion criteria were: features of nephritis, secondary NS, as well as those admitted for diagnostic renal biopsy and infusion therapy (cyclophosphamide or rituximab). The study was approved by Institutional Ethics Committee.

Based on previous studies,^6^^-^ 8 the average incidence of major infections in children with NS was assumed to be 35%. Sample size was calculated by the formula 4p (1-p)/d^2^, where p is the prevalence of major infections and d is the precision. A sample size of 91 children was calculated at 95% confidence interval and 7% precision (d). Assuming a 10% loss to follow up, we planned to enroll a minimum of 100 children.

Data were analyzed using SPSS version 23. Incidence of major infections was measured as a proportion of children diagnosed with major infections out of total episodes of hospitalizations with NS. Independent sample t-test and Chi square or Fischer’s exact tests were used to test the significance of difference between two means and proportions respectively. Mann-Whitney U-test was used to test the significance of difference between two medians, where data were skewed. Risk factors for infections were analyzed by logistic regression analysis.

## RESULTS

**Figure 1:**
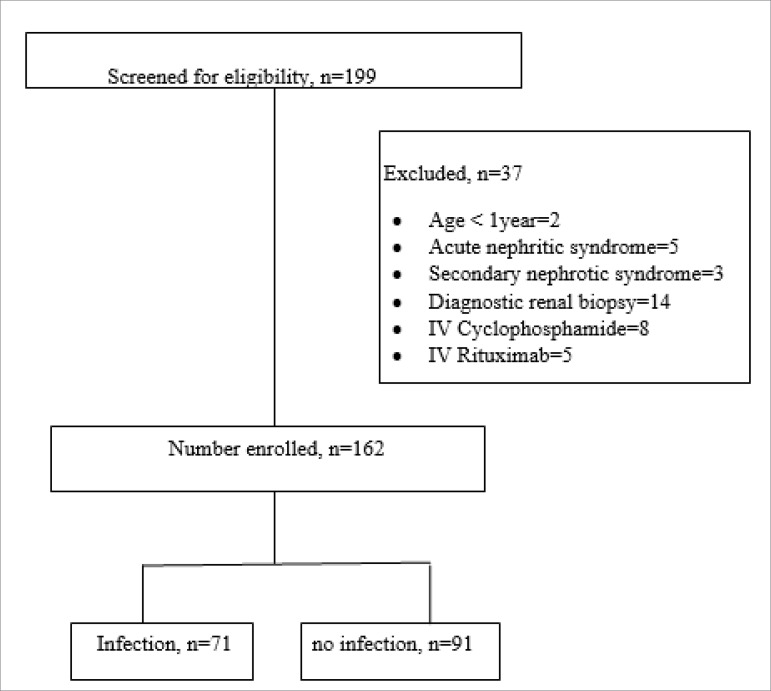
Study flow chart.

A total of 199 episodes of hospitalizations with diagnosis of NS were screened, out of which 37 were excluded. Finally, 148 children with 162 episodes of hospitalizations were enrolled (Figure 1). Baseline characteristics of the study population are depicted in Table 1. Indications for hospitalization were isolated anasarca (n = 81), anasarca with suspected infection (n = 59), suspected infection without anasarca (n = 12), hypovolemia (n = 7), tetany (n = 2), and hypertensive encephalopathy (n = 1). None of the children had received pneumococcal vaccine in the past. There were four (2.5%) deaths in our study population, all due to multi-organ failure resulting from major infections.

**Table 1:** Baseline characteristics of study population

Parameters	(n = 162)
Age in years, means and SD	5.3 ± 3.0
Age in years, median (IQR)	4.5 (3-8)
Age of onset of disease (years)	3.5 ± 2.3
Duration of nephrotic syndrome (years), median, IQR	1 (0-2.5)
Male, n (%)	86 (53%)
Type of NS, n (%)	
Initial episode	49 (30.2)
IFRNS	33 (20.4)
FRNS/ SDNS	50 (31)
SRNS	30 (18.5)
Remission status	
Remission	11 (7)
Relapse	102 (63)
Initial episode	49 (30)
Treatment received	
No treatment	36 (22.2)
Only prednisolone	88 (54.3)
Prednisolone with Levamisole	6 (3.7)
Prednisolone with cyclosporine	15 (9.3)
Prednisolone with cyclophosphamide	12 (7.4)
Prednisolone with MMF	3 (1.9)
Rituximab	2 (1.2)
Weight (Kg)	18.8 ± 9.1
Height (cm)	104 ± 20
Hb (g/dL)	11.5 ± 2.1
S. Creatinine (mg/dL)	0.58 ± 0.3
S. Albumin (g/dL)	1.5 ± 0.5
S. Cholesterol (mg/dL)	473 ± 151
Types of infections, n (% out of total number of infections)	
Peritonitis	17 (24.2)
Pneumonia	13 (18.5)
UTI	11(15.7)
Cellulitis	10 (14.2)
Acute diarrhea	4 (5.7)
Typhoid	3 (4.3)
Hepatitis	4 (5.7)
Tuberculosis	2 (2.8)
Meningitis	2 (2.8)
Varicella	1 (1.4)
Measles	1 (1.4)
Malaria	1 (1.4)
Sepsis	2 (2.8)
Death, n (%)	4 (2.5)
Duration of hospital stay (days)	10 ± 6.8

IQR: interquartile range; NS: nephrotic syndrome; IFRNS: Infrequently relapsing nephrotic syndrome; FRNS: frequently relapsing nephrotic syndrome; SDNS: steroid dependent nephrotic syndrome; SRNS: steroid resistant nephrotic syndrome; MMF: mycophenolate mofetil; UTI: urinary tract infection.

Baseline demographic, clinical, and laboratory characteristics of admitted children with and without infections are shown in Table 2. Age, gender, duration of NS, remission status, type of NS, immunosuppressive treatment, and biochemical parameters were not significantly different between the two groups. Duration of hospital stay in children with infection was significantly higher in comparison to children without infection (12 versus 8 days, *p* < 0.001).

**Table 2:** Baseline clinical and hematological characteristics in nephrotic children with and without.

Parameters	Infection (n = 71)	Without infection (n = 91)	*p* value; RR, 95% CI
Age (years)	5.4 ± 3.1	5.2 ± 2.9	0.67
Age of onset of disease (years)	3.4 ± 2.2	3.6 ± 2.3	
Duration of nephrotic syndrome (years), median IQR	1 (0.5-3)	0.8 (0-2)	0.46
Male, n (%)	38 (53%)	48 (53%)	
Type of NS, n (%)			0.49
Initial episode	17 (24)	32 (35)	
IFRNS	16 (23)	17 (19)	
FRNS/SDNS	23 (32)	27 (30)	
SRNS	15 (21)	15 (16)	
Remission status			0.30
Remission	6 (9)	5 (6)	
Relapse	48 (67)	54 (59)	
Initial episode	17 (24)	32 (35)	
Treatment received			0.09
No treatment	13 (19)	23 (24)	
Only prednisolone	41 (57)	47 (52)	
Prednisolone with Levamisole	0 (0)	6 (6.6)	
Prednisolone with cyclosporine	9 (13)	5 (5.5)	
Prednisolone with cyclophosphamide	6 (9)	6 (6.6)	
Prednisolone with MMF	1 (2)	2 (2.2)	
Rituximab	1 (2)	2 (2.2)	
Weight (Kg)	19.1 ± 9.4	18.6 ± 9	0.79
Height (cm)	103.6 ± 20	104 ± 20	0.84
Hb (g/dL)	11.6 ± 2.2	11.5 ± 2	0.66
S. Creatinine (mg/dL)	0.5 ± 0.3	0.6 ± 0.3	0.23
S. Albumin (g/dL)	1.5 ± 0.5	1.6 ± 0.5	0.28
S. Cholesterol (mg/dL)	447 ± 145	488 ± 133	0.06
Death, n (%)	4 (5.6)	0	0.03*; 2.4 (1.9-2.8)
Duration of hospital stay (days)	12 ± 8	8 ± 5	0.001*

RR: relative risk; IQR: interquartile range; NS: nephrotic syndrome; IFRNS: Infrequently relapsing nephrotic syndrome; FRNS: frequently relapsing nephrotic syndrome; SDNS: steroid dependent nephrotic syndrome; SRNS: steroid resistant nephrotic syndrome; MMF: mycophenolate mofetil.

* *p* value significant.

There were 71 episodes of major infections out of 162 hospitalizations, amounting to 43.8% incidence of major infections in hospitalized children with NS. Out of 71 episodes of major infections, bacterial peritonitis (n = 17, 24%), pneumonia (n = 13, 18%), urinary tract infection (n = 11, 15%), and cellulitis (n = 10, 14%) accounted for the majority (71%), followed by acute diarrhea (n = 4), acute viral hepatitis (n = 4), tuberculosis (n = 3), typhoid (n = 3), measles, varicella, malaria, and sepsis (n = 1 each). *Streptococcus pneumoniae* was the predominant organism isolated from blood and ascitic fluid (n = 9, 8 in blood and one in ascitic fluid). *E. coli* was the commonest organism isolated from urine (n = 7), followed by *Enterococcus faecium* (n = 2), *Klebsiella* (n = 1) and *Proteus* (n = 1). *Nocardia* and *Cryptococcus neoformans* were isolated from pleural and cerebrospinal fluid respectively from one child each (Table 3).

**Table 3:** Microorganism growth pattern in major infections in children with nephrotic syndrome

Culture site	Samples screened	Sample positive for growth, n (%)	Organism identified, n (%)
Blood	148	12 (8)	Streptococcus pneumoniae: 8 (66)
			Salmonella typhi: 3 (25)
			Pseudomonas: 1(9)
Urine	85	11 (13)	E. coli: 7 (64)
			Enterococus fecium: 2 (18)
			Klebsiella: 1 (9)
			Proteus: 1 (9)
Ascitic fluid	34	1 (3)	Streptococcus pneumoniae, 1 (9)
Pleural fluid	1	1	Nocardia, 1 (9)
CSF	2	1	Cryptococcus Neoformans,1 (9)

CSF- cerebrospinal fluid.

On logistic regression analysis, serum albumin < 1.5 g/dL was found as the only risk factor for major infections (OR 2.6; 95% CI, 1.2-6; *p* = 0.01) as well as peritonitis (OR 29; 95% CI, 3-270; *p* = 0.003). Age, gender, duration of disease, types of NS, immunosuppressive therapy and high serum cholesterol were not associated with increased risk of major infections and peritonitis (Table 4).

**Table 4:** Risk factors for major infections and peritonitis in children with nephrotic syndrome

Risk factors for infection			
Parameters	Odds Ratio (OR)	95% CI	*p* value
Male	1.3	0.6-3	0.45
Age	1	0.8-1.3	0.58
Duration of disease	1	0.8-1.2	0.78
Serum Albumin < 1.5 g/dL	2.6	1.2-6	0.01*
Serum Cholesterol > 500 mg/dL	0.6	0.2-1.3	0.22
Platelets > 500 cells/mm^3^	0.8	0.4-1.8	0.66
FRNS/SDNS	4.5	0.8-26	0.09
IFRNS	5	0.8-32	0.08
SRNS	6.6	0.9-46	0.06
Immunosuppressant therapy	0.3	0.05-2	0.22
Risk factors for peritonitis			
Parameters	Odds Ratio (OR)	95% CI	*p* value
Male	2.8	0.7-10.3	0.10
Age	0.9	0.7-1.2	0.80
Duration of disease	1.2	0.9-1.8	0.18
Serum Albumin < 1.5 g/dL	29	3-270	0.003*
Serum Cholesterol > 500 mg/dL	0.2	0.05-1.2	0.08
Platelets > 500 cells/mm^3^	2.1	0.6-8.3	0.25
FRNS/SDNS	1.5	0.2-19	0.74
IFRNS	3.1	0.2-42	0.38
SRNS	11.1	0.8-157	0.07
Immunosuppressant therapy	1.8	0.08-40	0.71

IFRNS: Infrequently relapsing nephrotic syndrome; FRNS: frequently relapsing nephrotic syndrome; SDNS: steroid dependent nephrotic syndrome; SRNS: steroid resistant nephrotic syndrome; CI: confidence interval.

## DISCUSSION

In our study, incidence of major infections in hospitalized children with NS was 43.8%, peritonitis being the commonest infection, followed by pneumonia and UTI. Serum albumin level less than 1.5 g/dL was the only independent risk factor for major infections including peritonitis. Duration of hospital stay was significantly higher in children with infections in comparison to without infection. There were four deaths (2.5%) in our study, all due to sepsis with multiorgan failure.

Major infections in children with NS have been reported from different parts of India and neighboring countries, with incidence varying from 20-38%.^3^^-^ 8 The relatively higher incidence of infection in our study population can be explained by referral bias and high index of clinical suspicion for infections in these children. In contrast, studies where minor infections were included as well 9 ^-^ 15, like upper respiratory tract infections, reported very high incidence of infection varying from 76% to 84%.

Peritonitis (24%) was the commonest infection in our study, similar to studies from other parts of the country.^4^^-^ 8 Studies have shown incidence of peritonitis in childhood NS ranging from 2.6-26%.^7^^-^ 9 ^,^ 13 Differently from our study, where *Streptococcus pneumoniae* was the only organism isolated from children with peritonitis, Senguttuvan et al.^7^ observed *E.coli* and *Klebsiella* as predominant organisms in peritonitis. None of the children in our study were immunized with pneumococcal vaccine. Given that pneumococcal vaccine is not included in our national immunization schedule, the majority of children coming to public sector hospitals are at risk of invasive pneumococcal diseases. However, revised guidelines on the management of NS from Indian Pediatric Nephrology Group suggest that all children with NS should receive vaccination against pneumococcal infections.^12^

In our study, UTI was the 3^rd^ most common infection, comprising 15% of all major infections. In contrast, studies on infections in NS from two different parts of India reported UTI as the commonest infection, with incidence varying from 13.7 to 46%.^3^^,^ 7 In another study from Saudi Arabia, UTI was the most common major infection, comprising 25% of total infections.^14^ In one of the largest retrospective analysis in children with NS to determine the incidence of UTI, 15% of children were found to have UTI, with more than 50% being asymptomatic and diagnosed as a part of screening investigations for relapse and non-response.^15^ This highlights the importance of screening for UTI in all children with NS with relapse or non-response to steroids, as symptoms may be masked because of anti-inflammatory action of steroids.

Very few studies have assessed risk factors for major infections in children with NS. In concordance with the literature, we found hypoalbuminemia as a risk for peritonitis in our study.^2^^,^ 3 ^,^ 13 Severity of hypoalbuminemia serves as a marker for urinary loss of immunoglobulins and complement factors required for opsonization, phagocytosis, and host defense. We did not find hypercholesterolemia as a risk factor for infection, in contrast to a study from south India,^8^ where serum cholesterol > 400 mg/dL was found to be an independent risk factor for peritonitis. In contrast to earlier studies,^3^^,^ 8 ^,^ 11 we did not observe a higher risk of infection in children suffering from more severe types of NS in comparison to initial episodes. Senguttuvan et al,^7^ showed higher risk of infection in children receiving a combination of prednisolone and cyclophosphamide. However, we did not find increased risk of infection in children receiving prednisolone alone or in combination with any other immunosuppressive agent in comparison to no treatment, confirming that these children remain in a state of immunosuppression and increased risk of infection irrespective of immunosuppressive therapy.

In our study, four children (2.5%) died of sepsis with multiorgan failure. The International Study of Kidney Disease in Children (ISKDC) followed almost 389 children with minimal change disease for 5-10 years and reported ten deaths, of which six were due to infection.^16^ In contrast, Srivastava et al.^4^ reported a very high death rate with 13% of children dying of infection, mostly within 24 hours of admission, indicating fulminant nature of infections associated with NS. The fewer deaths in our study can be explained by early presentation, high index of suspicion for infections and prompt institution of treatment.

To conclude, infections are common in hospitalized children with NS resulting in significant morbidity and mortality. Hypoalbuminemia was an independent risk factor for major infections, including peritonitis. As pneumococcus was the most prevalent cause for infections in our study population, attention should be paid to pneumococcal immunization in children with NS. These children should receive recommended doses of pneumococcal conjugate vaccine (PCV-13), followed by pneumococcal polysaccharide vaccine (PPSV-23) early in the course of disease.^17^

Limitations of our study include not measuring serum immunoglobulin as well as complement levels. Our study did not have adequate power for assessing risk factors for infection. Adequately powered studies with larger sample sizes are needed to assess risk factors for major infections and peritonitis in nephrotic children.
